# Plasma‐Strengthened Lithiophilicity of Copper Oxide Nanosheet–Decorated Cu Foil for Stable Lithium Metal Anode

**DOI:** 10.1002/advs.201901433

**Published:** 2019-08-15

**Authors:** Jingyi Luan, Qi Zhang, Hongyan Yuan, Dan Sun, Zhiguang Peng, Yougen Tang, Xiaobo Ji, Haiyan Wang

**Affiliations:** ^1^ Hunan Provincial Key Laboratory of Chemical Power Sources College of Chemistry and Chemical Engineering Central South University Changsha 410083 P. R. China

**Keywords:** copper oxide nanosheets, lithiophilicity, lithium metal anodes, lithium‐ion batteries, nitrogen plasma regulating

## Abstract

Lithium metal is the most ideal anode for next‐generation lithium‐ion batteries. However, the formation of lithium dendrites and the continuous consumption of electrolyte during cycling lead to a serious safety problems. Developing stable lithium metal anode with uniform lithium deposition is highly desirable. Herein, a nitrogen plasma strengthening strategy is proposed for copper oxide nanosheet–decorated Cu foil as an advanced current collector, and deep insights into the plasma regulating mechanism are elaborated. The plasma‐treated electrode can maintain a high coulombic efficiency of 99.6% for 500 cycles. The symmetric cell using the lithium‐plated electrode can be cycled for more than 600 h with a low‐voltage hysteresis (23.1 mV), which is much better than those of electrodes without plasma treatment. It is well confirmed that this plasma‐induced nitrogen doping method can provide sufficient active sites for lithium nucleation to enhance the stability of lithium deposition on copper oxide nanosheets decorated on Cu foil and improve the electrical conductivity to greatly reduce the overpotential of the lithium nucleation, which can be extended to other modified current collectors for stable lithium metal anode.

## Graphical abstract

**A nitrogen plasma strengthening strategy** is proposed for copper oxide nanosheet–decorated Cu foil as an advanced current collector. The plasma‐induced nitrogen doping can provide sufficient active sites for lithium nucleation to enhance the stability of lithium deposition. This strategy can be extended to other modified current collectors for stable lithium metal anode.

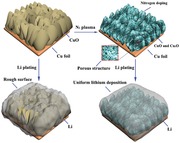

## 1 Introduction

Lithium‐ion batteries (LIBs) have been extensively used in daily life for portable electronics and electric vehicles due to their high energy density, long lifespan, and environment benignity.^1^ However, their electrochemical performances are generally limited by the lacking of advanced electrode materials, especially the anode materials.^2^ Graphite is the most prevailing anode material in commercialized LIBs. However, its relatively low theoretical capacity (372 mAh g^−1^) cannot meet the requirements of next‐generation high‐energy‐density LIBs.^3^ The zero‐strain Li_4_Ti_5_O_12_ with excellent cycling stability is also criticized for its low energy density because of the high working plateau at ≈1.55 V (vs Li/Li^+^). Therefore, it is still a big challenge to develop new anode materials with high specific capacity, good cyclic stability, and pronounced rate capability for LIBs.

Lithium metal has been supposed as the most promising anode for next‐generation high‐power and high‐energy‐density batteries due to the advantages of high theoretic gravimetric capacity (3860 mAh g^−1^) and lowest electrochemical potential (−3.04 V vs the standard hydrogen electrode) comparing with other anodes.^4^ However, lithium metal is usually deposited randomly with unpredictable shape and large volume change during repeated Li plating/stripping, resulting in the formation of Li dendrites.^5^ It is an inevitable hurdle, which leads to short cycle life and poor safety characteristics of lithium metal batteries.^6^ On the one hand, lithium has high electrochemical activity and is easy to react with organic electrolytes and form solid electrolyte interphase (SEI).^7^ The formed SEI layer can be easily fractured by the moss‐like Li dendrites, which will lead to the exposure of fresh Li in the electrolyte and the formation of new SEI layer. Thus, the accelerated depletion of both electrolyte and Li metal electrode is the inherent cause of the low coulombic efficiency (CE). On the other hand, the accumulation of “dead” Li can penetrate separators and generate internal short circuit together with a safety hazard.^8^

To resolve the Li dendrite issue mentioned above, many available efforts have been devoted in recent years to suppressing, preventing, or blocking the dendrites.^9^ Generally, three important strategies can be summarized toward the lithium metal electrode.^7^ The first one is to inhibit the breaking of ubiquitous SEI films. For examples, massive self‐healing of the dendrites causes the large surface migration of lithium, and this surface diffusion heals the lithium dendrites;^10^ The control of SEI film broken regions can reduce the releasing stress via SEI film sewing.^11^ The external field influencing route can stabilize the lithium metal anodes and suppress the growth of Li dendrites.^12^ The second is utilizing a solid electrolyte to preclude dendrite proliferation in order to avoid the reaction between Li metal and liquid electrolyte.^13^ The third is to confine the lithium within a 3D structure and thus induces the stable plating of Li ions to prevent the formation of Li dendrites, resulting in long‐term stable cycling.^14^ Therefore, a series of substrate materials modified by metal oxides (e.g., TiO_2_,^15^ ZnO,^16^ and CuO^17^) were proposed as current collectors for Li metal anode due to their superior lithiophilicity for facilitating the lithium nucleation. However, most metal oxides have low electrical conductivity owing to intrinsic semiconductor characteristic.^18^ Therefore, improving the electrical conductivity of outside metal oxides by some physical or chemical methods is essential. Nitrogen doping is a widely used strategy to optimize the crystal structure of materials. However, some inefficient common methods such as calcination in N_2_ atmosphere and hydrothermal process with urea assistance cannot achieve a high‐concentrated nitrogen doping owing to their moderate treatment.^19^ N_2_ plasma, a kind of high‐energy‐ionized gas, can efficiently create deep nitrogen doping in the electrode materials and yield no chemical wastes, which may be a promising approach to modify the metallic oxide for enhancing the reactivity and electrical conductivity.^20^

In this work, we developed a novel and impressive plasma strengthening strategy to synthesize nitrogen‐doped porous copper oxide–decorated Cu foil for dendrite‐free lithium metal anode, where the Cu foil was used for both the current collector and the substrate for CuO growth. This plasma strengthening strategy can easily form a large number of active sites on the electrode surface by nitrogen doping, which could reduce lithium nucleation potential. Benefitting from the low lithium nucleation potential and regulation of plasma parameters, no lithium dendrites were generated during the repeated lithium deposition and the optimal plasma‐treated electrode could be cycled stably with a very high CE of 99.6%.

## 2 Results and Discussion

**Scheme 1:**
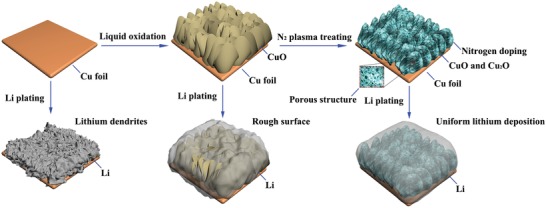
Schematic illustration of the plasma strengthening mechanism for CF@PN–CNS and the lithium plating process on the CF, CF@CNS, and CF@PN–CNS.

**Figure 1:**
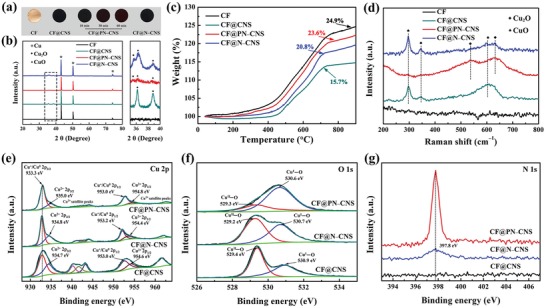
a) Photographs of CF, CF@CNS, CF@PN–CNS–10/30/60, and CF@N–CNS. b) XRD patterns of CF, CF@CNS, CF@PN–CNS, and CF@N–CNS (the rightward image is the magnified view of the marked part of the four samples). c) TG curves of CF, CF@CNS, CF@PN–CNS, and CF@N–CNS. d) Raman spectra of CF, CF@CNS, CF@PN–CNS, and CF@N–CNS. e) Cu 2p XPS spectrum of CF, CF@CNS, CF@PN–CNS, and CF@N–CNS. f) O 1s XPS spectrum of CF, CF@CNS, CF@PN–CNS, and CF@N–CNS. g) N 1s XPS spectrum of CF@CNS, CF@PN–CNS, and CF@N–CNS.

The plasma‐strengthening mechanism and the lithium‐plating process are illustrated in **Scheme** 1. The nitrogen‐doped porous copper oxides are obtained by liquid oxidation of bare Cu foil and the subsequent plasma treating. During the lithium deposition, the bare Cu foil may cause the growth of lithium dendrites owing to its poor lithiophilicity. By contrast, this plasma treatment can provide abundant bonding sites for lithium nucleation and form the porous structure, which tends to improve the lithium plating and migration. **Figure** 1a shows the photographs of CF, CuO nanosheet–decorated Cu foil (CF@CNS), plasma‐induced N‐doped CuO nanosheet–decorated Cu foil (CF@PN–CNS), and CF@N–CNS. The color changes of copper foils are observed owing to the different chemical treatment methods and processing time. The Cu foil turns from bright yellow to black after oxidized by (NH_4_)_2_S_2_O_8_. Moreover, with the increase of plasma treating time, the color of CF@PN–CNS changes to brick‐red gradually. In contrast to CF@CNS, CF@N–CNS maintains original black, and the color is barely changed. The X‐ray diffraction (XRD) patterns (Figure 1b) display the crystal phases of Cu, CuO, and Cu_2_O. As seen, all the diffraction peaks of the products are consistent well with the standard patterns of Cu (JCPDS No. 04–0836), CuO (JCPDS No. 45–0937), and Cu_2_O (JCPDS No. 05–0667).[qv: 2a] No other impurity peaks indicate the high purity of the samples. In CF@CNS, only CuO peaks exist. However, both CuO and Cu_2_O peaks are observed in CF@PN–CNS and CF@N–CNS, and the peaks of CF@PN–CNS have low intensity compared with those of CF@N–CNS, which reveals that the reduction degree of CuO to Cu_2_O is higher in CF@PN–CNS. Figure 1c displays thermogravimetry (TG) curves of four samples heating from 25 to 900 °C. The weight increment is assigned to the oxidation of Cu or Cu_2_O. CF@PN–CNS has a higher weight increment in the TG curve than CF@N–CNS due to the higher proportion of Cu_2_O existing, as mentioned above in Figure 1b. From the Raman spectra (Figure 1d), three peaks of CF@PN–CNS, CF@N–CNS, and CF@CNS located at about 297, 343, and 617 cm^−1^ are corresponding to the typical peaks of CuO. Two peaks at 535 and 620 cm^−1^ matching with CF@PN–CNS and CF@N–CNS can be indexed to Cu_2_O, which are in good agreement with the results of XRD and TG above.

X‐ray photoelectron spectroscopy (XPS) measurements are investigated for CF@PN–CNS, CF@N–CNS, and CF@CNS to examine the chemical composition and oxidization states. The wide scan XPS spectrum (Figures S1 and S2, Supporting Information) reveals the predominant existence of C, O, N, and Cu elements. Among these elements, N, Cu, and O elements are from the material and the C element is from the XPS instrument itself. No other heteroelements are detected. As illustrated in Figure 1e, the Cu 2p for CF@CNS displays the characteristic features for Cu^2+^ and Cu^0^. The peaks located at 934.7 and 954.6 eV correspond to Cu 2p_3/2_ and Cu 2p_1/2_ of Cu^2+^, while those at 933.3 and 953.0 eV are related to Cu 2p_3/2_ and Cu 2p_1/2_ of Cu^0^. Because the binding energies of the Cu^+^ and Cu^0^ states in the Cu 2p peaks are similar, it is difficult to distinguish them.[qv: 18b] There is a series of satellite peaks in the ranges of 940–946 and 960–965 eV for Cu 2p_3/2_ and Cu 2p_1/2_ in CuO, respectively, arising from electron‐correlation effects in the open Cu 3d shell (3d^9^). Almost no significant deviation is inspected from the peak position of CF@PN–CNS and CF@N–CNS in comparison to CF@CNS. But in CF@PN–CNS, it is obvious that the peak intensity of Cu^+^ and Cu^0^ is higher than that of Cu^2+^, which is quite opposite in CF@N–CNS and CF@CNS. It is ascribed to the stronger reduction effect of the N_2_ plasma process and a greater proportion of CuO reduced to Cu_2_O and Cu in CF@PN–CNS. As shown in Figure 1f, O 1s peak can be fitted into two peaks in CF@CNS. The peak at 529.4 eV corresponds to lattice oxygen within CuO, while that at 530.9 eV can be attributed to lattice oxygen in Cu_2_O.[qv: 19b] Owing to the better reduction effect of N_2_ plasma treating, the peak intensity of Cu^I^–O in CF@PN–CNS is higher than that of Cu^II^–O in CF@N–CNS and CF@CNS. The binding energy locating at 397.8 eV is assigned to N 1s in CF@PN–CNS and CF@N–CNS, revealing that the element of N was doped into the material in the preparation process (Figure 1g). As expected, by means of N_2_ plasma treating, a much higher percentage of N element doped in CF@PN–CNS is supported by a higher and sharper peak of N 1 peak in comparison with CF@N–CNS.

To further analyze the effect of plasma treating time on nitrogen doping, CF@CNS was processed under N_2_ plasma for 10 and 60 min (CF@PN–CNS–10 and CF@PN–CNS–60). Combining the results with the dataset of XRD patterns (Figure S3, Supporting Information), TG curves (Figure S4, Supporting Information), Raman spectra (Figure S5, Supporting Information), and XPS spectrum (Figure S6, Supporting Information) of CF@PN–CNS–10 and CF@PN–CNS–60, the proportion of CuO in CF@PN–CNS–60 is lower than CF@PN–CNS–10. The stronger reduction of CuO to Cu and Cu_2_O in CF@PN–CNS–60 is resulted from the longer N_2_ plasma treating. Additionally, the high‐energy particles in plasma attacking the surface may result in further loss of CuO with the extended reacting time.[qv: 20a] N1 XPS in Figure S6 (Supporting Information) indicates that plasma treating for 30 min in CF@PN–CNS has the best nitrogen‐doping effect as it has the highest characteristic peak compared to CF@PN–CNS–10 and CF@PN–CNS–60.

**Figure 2:**
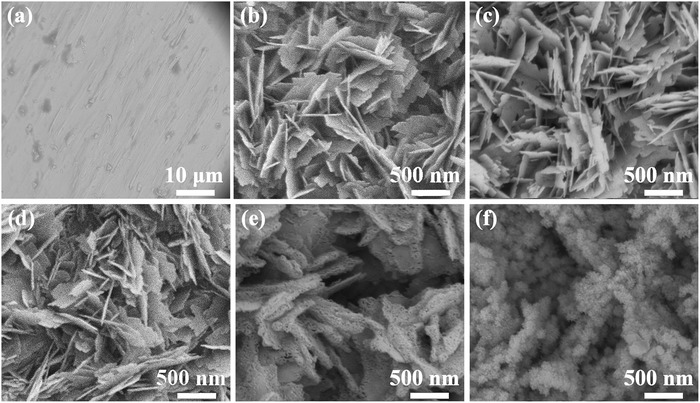
SEM images of a) CF, b) CF@CNS, c) CF@N–CNS, d) CF@PN–CNS–10, e) CF@PN–CNS, and f) CF@PN–CNS–60.

The morphologies of all samples are monitored by the scanning electron microscopy (SEM) in **Figure** 2. For comparison, the SEM image displays the smooth surface of pure copper foil (Figure 2a). When the copper foil was oxidized by (NH_4_)_2_S_2_O_8,_ the 3D CuO nanosheets are attached on the copper foil successfully (Figure 2b). CF@N–CNS and CF@PN–CNS–10 (Figure 2c,d) still maintain the original morphology, while different changes take place in structure and morphology in CF@PN–CNS and CF@PN–CNS–60 (Figure 2e,f). CF@PN–CNS shows porous nanosheets by means of plasma processing for 30 min. Extending the treating time to 60 min, the nanosheets turn into nanoparticles totally in CF@PN–CNS–60 owing to being attacked by highly active particles in plasma for a long time.[qv: 19b] As seen from the section view of SEM (Figure S7, Supporting Information), CF@N–CNS exhibits the tightest contact between CuO nanosheets and copper foil compared with other samples, which is beneficial for maintaining the stable structure during cycling. For further investigating the structure evolution and crystal growth of CF@CNS and CF@PN–CNS, the interface details were observed by using transmission electron microscopy (TEM) and high‐resolution TEM (HRTEM) in Figure S8 (Supporting Information). Figure S8a,c (Supporting Information) shows the overall morphology of nanosheets, which were obtained by ultrasonic stripping. As depicted in Figure S8b (Supporting Information), the distinct lattice spacing of 0.232 nm matches well with Miller indices of (111) of CuO.^21^ Compared with CF@CNS in Figure S8d (Supporting Information), after plasma treating for 30 min, the lattice spacings of 0.255, 0.275, and 0.245 nm are consistent with (002) plane of CuO, (110) plane of CuO, and (111) plane of Cu_2_O, respectively.^22^

**Figure 3:**
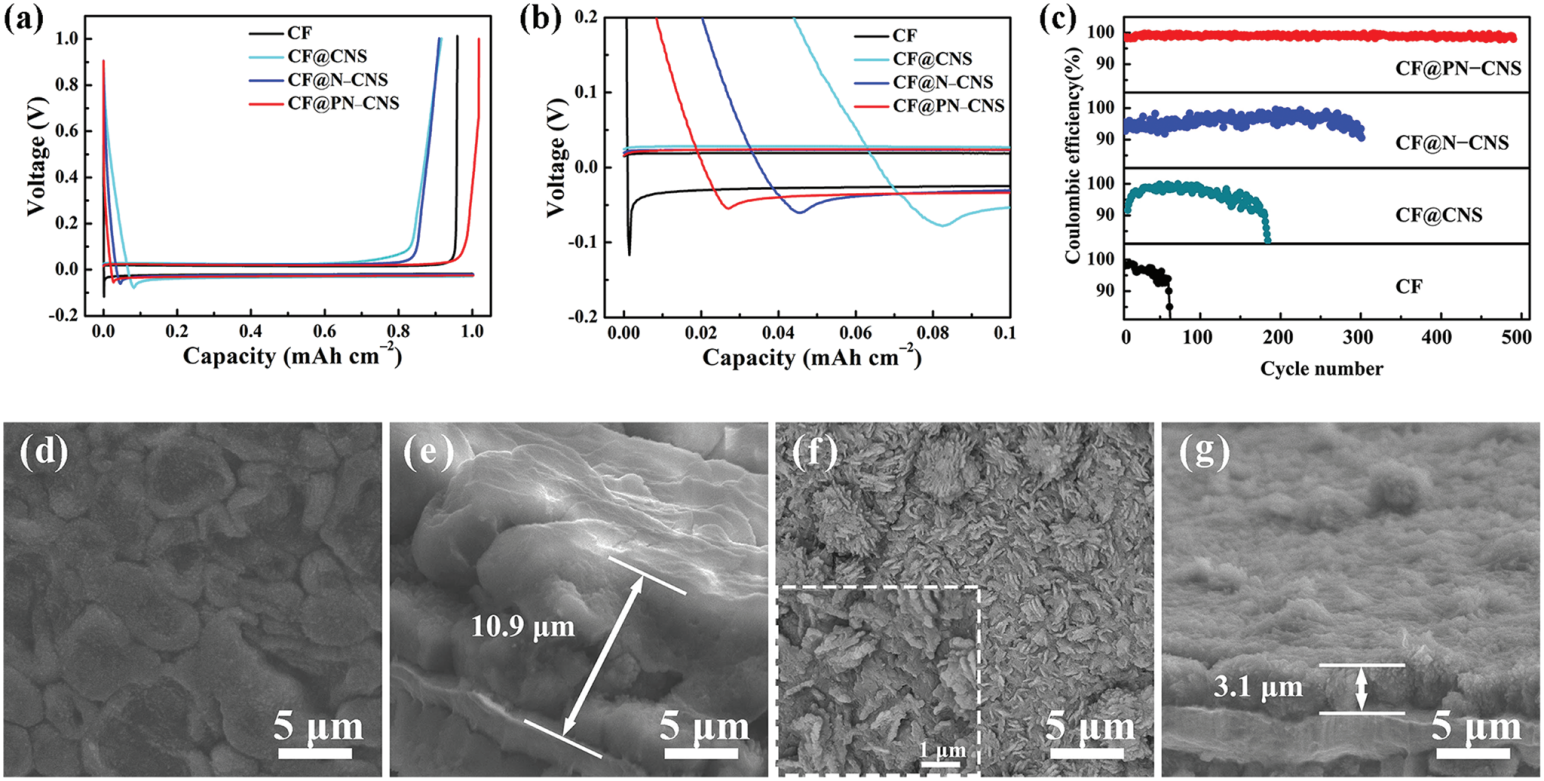
a) Charge–discharge curves of the plating and stripping processes at 1 mA cm^−2^ in the first cycle. b) Magnified image of the charge–discharge curves during initial lithium plating. c) The comparison of CE at 1 mA cm^−2^ with a specific capacity of 1 mAh cm^−2^. d–g) SEM images of CF@PN–CNS after the first lithium plating and the first lithium stripping.

To investigate the superiority of plasma‐induced nitrogen doping and 3D porous copper oxide nanosheets on lithium stripping/plating behavior, the charge–discharge curves of the plating and stripping processes at 1 mA cm^−2^ in the first cycle are given in **Figure** 3a and Figure S9a (Supporting Information), respectively. The nucleation of lithium in the first discharge cycle is highly valued, which determines lithium deposition that influences the stability of the long‐term cycle.^23^ Lithium nucleation overpotential, interpreted as the difference between the voltage platform and the voltage dip, is used to measure the uniformity of lithium.^24^ As shown in Figure 3b, the lithium nucleation overpotentials on CF and CF@CNS are figured out to be 97 and 46.4 mV, respectively. It suggests that the better lithiophilicity of CuO in CF@CNS could obviously decrease the energy barrier of lithium nucleation at another interface to induce uniform deposition on the surface.[qv: 17a] By means of nitrogen doping, the electrical conductivities of CF@N–CNS and CF@PN–CNS were significantly improved, which overcame the drawback of CuO as a kind of semiconductor. Besides, the increase of active sites for lithium nucleation reduced the resistance of heterogeneous nucleation.^25^ Therefore, the lithium nucleation overpotentials of CF@N–CNS and CF@PN–CNS are much lower, calculated as 40.1 and 35.8 mV, respectively. As seen in Figure S9b (Supporting Information), the lithium nucleation overpotentials of 38.0 and 37.4 mV correspond to CF@PN–CNS–10 and CF@PN–CNS–60, respectively. It certifies that 30 min is the best plasma treating time in CF@PN–CNS owing to the highest proportion of nitrogen doping and lowest lithium nucleation overpotential. To further demonstrate the lithiophilicity of CF@PN–CNS, the molten lithium wetting capability is illustrated in Figure S10 (Supporting Information). As seen clearly, the molten lithium can be uniformly spread out on CF@PN–CNS, while it forms a droplet on Cu surface.

Figure 3c shows the CEs of CF, CF@CNS, CF@N–CNS, and CF@PN–CNS cycled at 1 mA cm^−2^ with a specific capacity of 1 mAh cm^−2^. CF@PN–CNS shows a high CE of 98.7%, which is higher than CF (96.0%), CF@CNS (91.6%), CF@N–CNS (95.4%), CF@PN–CNS–10 (93.2%, Figure S9b, Supporting Information), and CF@PN–CNS–60 (94.8%, Figure S9b, Supporting Information) in the first discharge cycle, revealing superior performance in plating/stripping lithium. Combining SEM images of CF@PN–CNS (Figure 3d–g) after the first lithium plating and stripping, it is noted that the lithium is plated on the surface smoothly and uniformly with no dendrites. The structure of 3D porous nanosheets was maintained well after the first lithium stripping, and the thicknesses of the electrodes were 10.9 and 3.1 µm corresponding to the lithium plating and stripping processes, respectively. The thinnest layer of CF@PN–CNS indicates the best lithiophilicity and the highest CE in the first discharge cycle. In contrast, as shown in Figure S11 (Supporting Information), other samples demonstrate the rough surface and thicker lithium plating and stripping layers derived from inferior lithium affinity.

The CE of Cu foil electrode rapidly decreases in 60 cycles and is even lower than 50% after 70 cycles, which should be due to the fact that irregular and uncontrollable Li dendrites are generated during the reaction between Li and the liquid electrolyte. Meanwhile, continuous formation of SEI on the electrode resulting in the consumption of Li seriously impeded the migration of Li ions. The structure of 3D nanosheets in CF@CNS inhibited the growth of Li dendrites partly. The lithiophilic CuO can effectively reduce the local current density, and the enhanced specific surface area can also alleviate the uneven deposition on the Cu surface.[qv: 17a] Thus the CE and cycle life could be slightly improved. To our surprise, CF@N–CNS and CF@PN–CNS show excellent long‐term cycling performance. Especially for CF@PN–CNS, the CE is stabilized at 99.6% after 500 cycles while that of CF@N–CNS begins to decay after 260 cycles. The CE of CF@PN–CNS here is also higher than other reported current collectors, such as MnO_2_–Cu,^26^ ZnO‐decorated 3D hierarchical porous carbon,^27^ and graphene‐anchored Cu foam.^28^ A more detailed comparison of electrochemical performance for previously reported current collectors is given in Table S1 (Supporting Information), from which we can see CF@PN–CNS demonstrates superior cycling stability. Except for lithiophilic CuO, Cu_2_O is also characterized by XRD, Raman, and XPS results above, which can significantly reduce the lithium nucleation overpotential due to the superior lithiophilicity.^29^ The optimized 3D porous nanosheet structure can not only completely avoid the formation of lithium dendrites, but also promote the transport of the lithium ions. Meanwhile, as mentioned in N 1s XPS spectrum of CF@CNS, CF@PN–CNS, and CF@N–CNS, the best effect of nitrogen doping was acquired by plasma treating for 30 min in CF@PN–CNS with greatly improved electrical conductivity. More significantly, plentiful active sites for the lithium nucleation were exposed owing to the perfect quantity of nitrogen doping by plasma treating and thus induced the lithium nucleation to distribute evenly and uniformly.^30^ As depicted in Figure S12 (Supporting Information), CF@PN–CNS–10 and CF@PN–CNS–60 with the introduction of nitrogen also have better long‐term cycling performance compared with CF@CNS, but it is still much inferior to CF@PN–CNS. Therefore, the plasma process with 30 min is the best condition for structural regulating and nitrogen doping.

**Figure 4:**
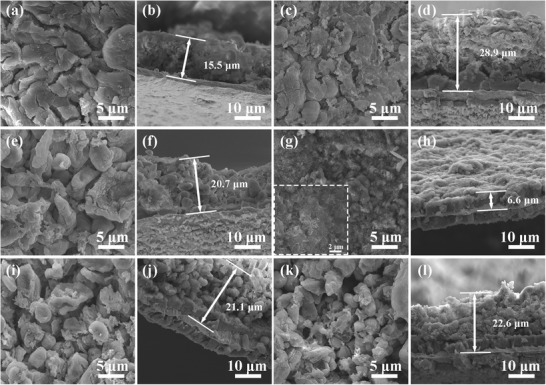
SEM images of a,b) CF, c,d) CF@CNS, e,f) CF@N–CNS, g,h) CF@PN–CNS–10, i,j) CF@PN–CNS, and k,l) CF@PN–CNS–60 after 100th lithium stripping.

**Figure 5:**
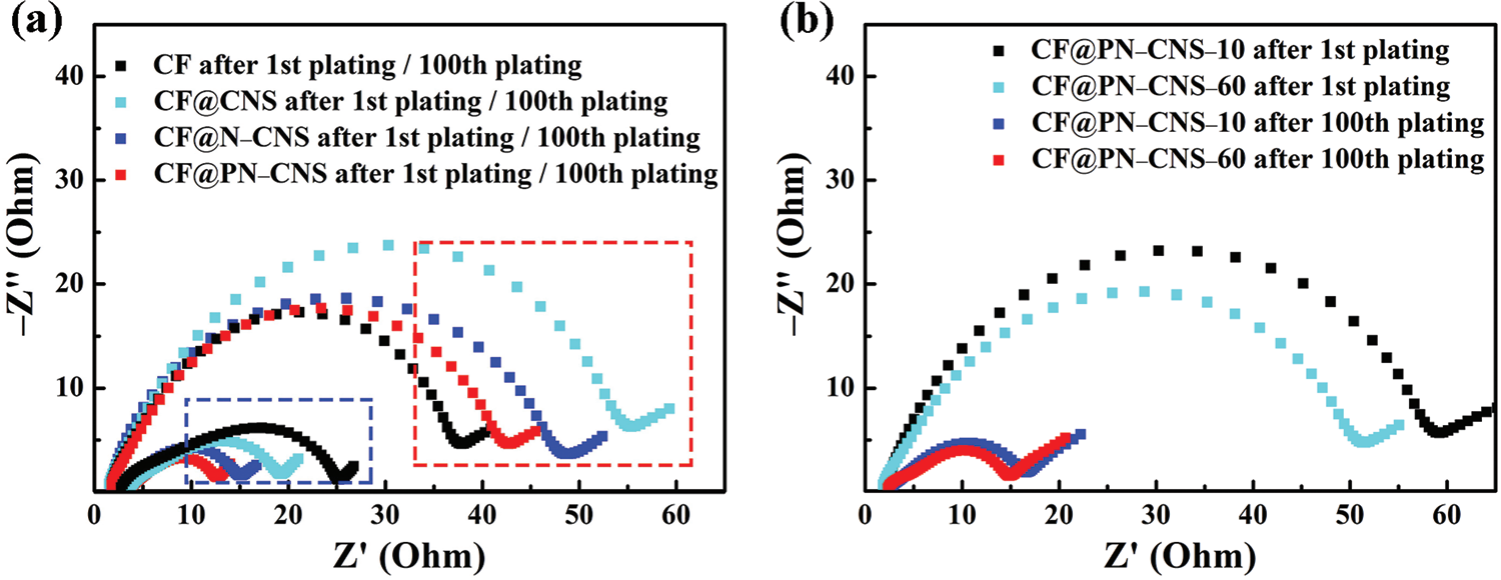
a) EIS plots of CF, CF@CNS, CF@N–CNS, and CF@PN–CNS after 1st (outlined by the dashed red box) and 100th (outlined by the dashed blue box) cycles. b) EIS plots of CF@PN–CNS–10 and CF@PN–CNS–60 after 1st and 100th cycles.

In order to further validate the capacity stabilization mechanism of CF@PN–CNS and poor long‐term cycling performance of other samples, the morphologies of these electrodes after 100th lithium stripping are compared in **Figure** 4. As exhibited in Figure 4a,b, many cracks on the electrode with rough surface can be observed on CF owing to the poor cycling stability and a mass of lithium dendrites grew on the copper layer with 15.5 µm thickness, much higher than that of the first lithium plating, which is caused by the inferior lithiophilicity of CF and the nonuniform lithium deposition. No cracks exist on CF@CNS, CF@N–CNS, CF@PN–CNS–10, and CF@PN–CNS–60 (Figure 4c–f,i–l) due to the improvement of lithiophilicity. However, a certain amount of residual lithium still remains on the surfaces and is not stripped. The increased thickness (>20 µm) indicates the existence of dead Li compared with those of the first lithium plating. In Figure 4g,h, the bulk of lithium on the electrode migrates to the counter electrode, and the morphology of nanosheets in CF@PN–CNS is exposed totally. The increased thickness of CF@PN–CNS is not obvious compared with the first lithium plating. To our surprise, the thickness of CF@PN–CNS after 100th lithium stripping is almost unchanged, revealing the excellent cycling performance. Beyond that, the poor cycling stability of other samples is just the result of the accumulation of dead Li. The Nyquist diagram is an important measurement method to evaluate the interfacial resistance of Li^+^ migrating through SEI and the migration resistance to charge transfer on the electrode surface.^31^ **Figure** 5 demonstrates electrochemical impedance spectroscopy (EIS) of the samples after the 1st and 100th cycles. The impedance of CF@PN–CNS is the lowest among all the samples after the 100th cycle, confirming the great superiority of lithiophilic copper oxides and plasma‐induced nitrogen doping. In the first cycle, the good electrical conductivity leads to the lowest impedance of CF. Note that the electrodes after 100 cycles demonstrate much lower impedance in comparison with those after first cycle. Some probable reasons are involved. First, the electrode after one cycle would be not well activated; therefore, it shows large impedance. Second, the newly formed SEI film was unstable with increased impedance after first cycle compared to the 100th cycle. Third, the conversion reaction (CuO + 2Li^+^ + 2e^−^ → Li_2_O + Cu) yielded Cu species with much higher electronic conductivity, which would reduce the impedance of electrode after the CuO was completely converted.[qv: 17b]

Figure S13 (Supporting Information) demonstrates the cyclic voltammetry (CV) curves of all the samples with lithium counter electrodes in the initial three cycles. Among these, the minimum polarization is observed from the smallest distance between the oxidation peak and the reduction peak of CF@PN–CNS, which illustrates the great electrochemical performance from another aspect. Also, the best overlapping effect of different cycles in CF@PN–CNS further testifies its superior cycling stability as above.

**Figure 6:**
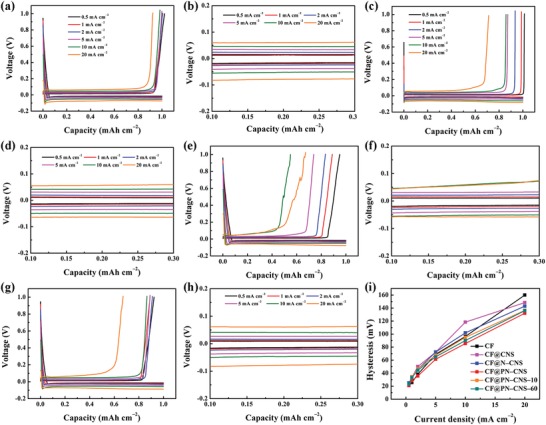
Plating/stripping profiles of a) CF@PN–CNS, c) CF, e) CF@CNS, and g) CF@N–CNS at various current densities. Magnified image of the plating/stripping profiles of b) CF@PN–CNS, d) CF, f) CF@CNS, and h) CF@N–CNS measured from 0.1 to 0.3 mAh cm^−2^. i) Voltage hysteresis comparison at different current densities.

**Figure** 6a,b displays the lithium plating/stripping profiles of CF@PN–CNS at various current densities from 0.5 to 20 mA cm^−2^ with a specific capacity of 1 mAh cm^−2^. With the increasing current density, the curves can maintain the steady voltage plateau and high CE of almost 100% at the current density lower than 10 mA cm^−2^. When it raises to 20 mA cm^−2^, a high CE of 93.2% can still be achieved. However, as shown in Figure 6c–f, the CEs of CF and CF@CNS decay severely, and the fluctuation of charging curves is obvious, which may be ascribed to the fewer active sites for lithium deposition and poor electrical conductivity of CuO, respectively.[qv: 17b] The CEs of CF@N–CNS (Figure 6g,h), CF@PN–CNS–10 (Figure S14a,c, Supporting Information), and CF@PN–CNS–60 (Figure S14b,d, Supporting Information) are relatively lower in comparison with CF@PN–CNS but much better than those of CF and CF@CNS owing to the slightly increased active sites for lithium nucleation. For further illustrating the excellent rate performance of CF@PN–CNS, the voltage hysteresis comparison at different current densities is demonstrated in Figure 6i. The voltage hysteresis of CF is the lowest among all the samples when the current density is increased from 0.5 to 2 mA cm^−2^, revealing the superior electrical conductivity. Fewer active sites for lithium nucleation lead to the formation of the lithium bulk on CF and rapid increase of the voltage hysteresis at high current density.^32^ Accordingly, it can be highlighted that plasma‐strengthening strategy is the vital requirement for the excellent rate performance, which forms abundant active sites for lithium nucleation and the 3D porous structure for inhibiting the growth of lithium dendrites.

**Figure 7:**
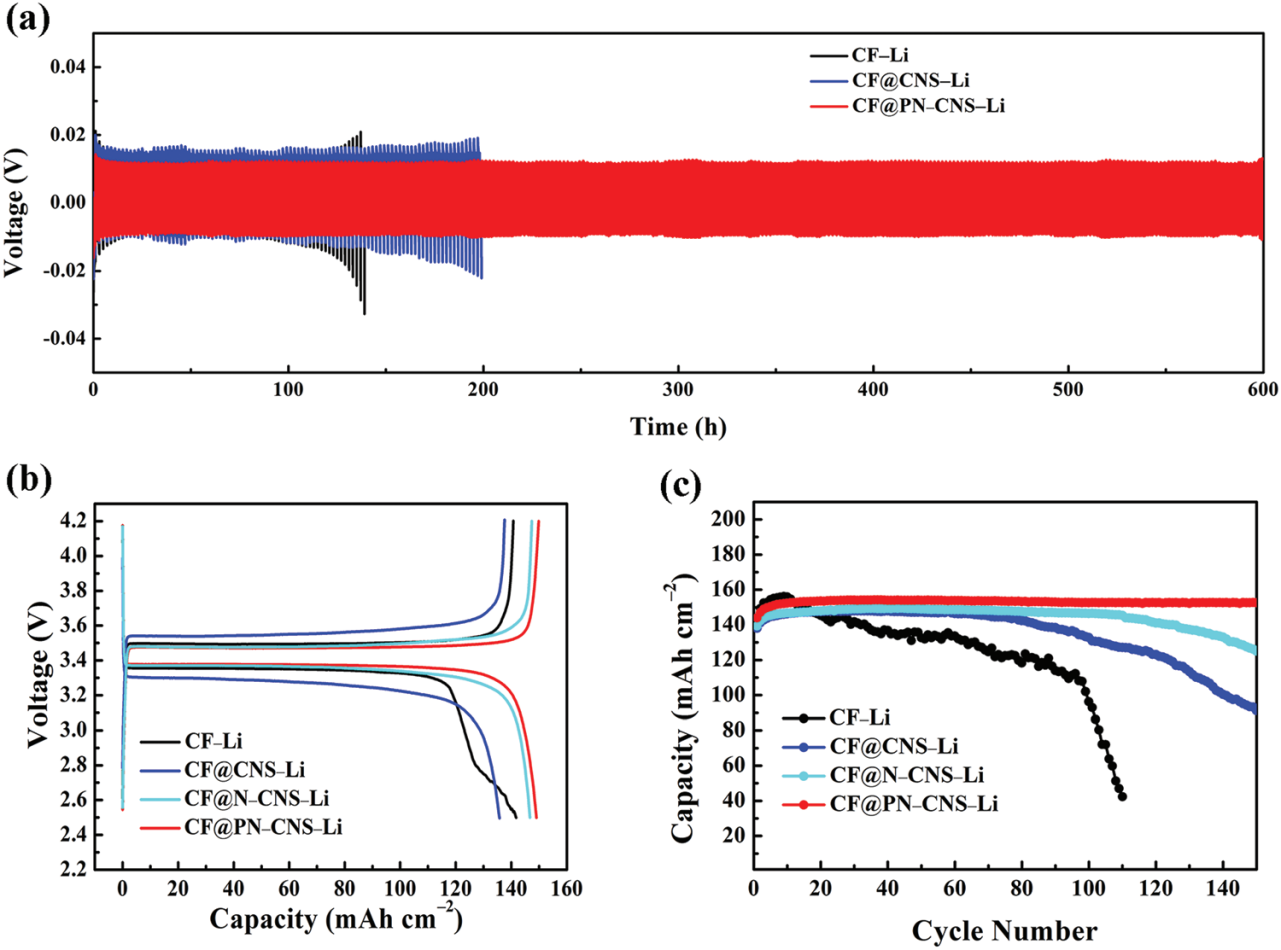
a) Galvanostatic plating/stripping profiles of CF–Li, CF@CNS–Li, and CF@PN–CNS–Li in symmetric cells. b) Charge–discharge profiles of full cells with LiFePO_4_ as the cathode and CF@PN–CNS–Li (CF–Li, CF@CNS–Li, and CF@N–CNS–Li) as the anode at 100 mA g^−1^. c) Cycling performance of full cells at 100 mA g^−1^.

The symmetric cells are assembled to investigate the long‐term cycling stability. As shown in the voltage–time curves (**Figure** 7a), CF@PN–CNS–Li can be continuously cycled over 600 h, demonstrating the prominent advantage of long‐term cycling performance. As for CF–Li and CF@CNS–Li, the voltage began to oscillate at about 100 and 180 h, respectively. CF@PN–CNS–Li can also maintain the lowest voltage hysteresis of 23.1 mV after repeating cycling. The superior lithiophilicity of copper oxides, the 3D porous structure, and the sufficient active sites for lithium nucleation synergistically ensure the stability and reversibility of CF@PN–CNS. To test the practicality, full cells were fabricated with commercial LiFePO_4_ (LFP) as the cathode. As indicated in Figure 7b and Figure S14a (Supporting Information), the lowest polarization and most stable platform of CF@PN–CNS–Li are observed compared with other samples in the charge–discharge profiles, demonstrating the significantly improved kinetics.^33^ In Figure 7c, CF@PN–CNS–Li/LFP can maintain 150 stable cycles with high specific capacity (152.6 mAh g^−1^), and slight capacity fading can be observed in CF@N–CNS–Li/LFP, while CF–Li/LFP and CF@CNS–Li/LFP exhibit fast capacity fading after 20 and 70 cycles, respectively.

## 3 Conclusion

In summary, nitrogen plasma was introduced to regulate the interface electrochemistry of the copper oxide nanosheet–modified Cu foil as the advanced current collector for lithium metal anode. Owing to the plasma treatment, nitrogen doping and abundant porosity in copper oxide nanosheets were easily achieved, leading to the greatly improved electrical conductivity and more active sites for lithium nucleation. In view of the above advantages, the uniform lithium dendrite‐free distribution contributed to the excellent lithium plating and stripping performance with the high CE of 99.6%. The symmetric cell of CF@PN–CNS–Li could cycle stably over 600 h with the small voltage hysteresis of 23.1 mV at 1 mA cm^−2^ for 1 mAh cm^−2^. When using commercial LiFePO_4_ as the cathode in full cells, the electrode of CF@PN–CNS exhibited excellent electrochemical performance and maintained the high discharge specific capacity after 150 cycles. This rational design of plasma‐induced nitrogen doping porous copper oxide nanosheets with highly stable and low‐cost lithium metal anode can expand the train of thought on plasma‐induced nitrogen doping and have more practical applications on the commercial anode current collectors.

## 4 Experimental Section

*Synthesis of CF@CNS*: CF@CNS was synthesized by a liquid oxidation method in an alkaline solution.[qv: 1c] All reagents were of analytical grade and used directly without purification. Typically, Cu foil was cut into a disk of 1.2 cm in diameter and then cleaned with water under ultrasonication for 10 min. After dried at 60 °C for 2 h, eight pieces of Cu disks were submerged in a 30 mL aqueous solution containing 1.5 g of NaOH and 0.34 g of (NH_4_)_2_S_2_O_8_ at 60 °C for 2 h. The products were washed with distilled water, then dried in air at 60 °C. The side of the Cu disk in contact with the solution turned black and the other side maintained the original bright yellow.

*Synthesis of CF@PN*–*CNS and CF@N*–*CNS*: CF@PN–CNS was synthesized by processing the as‐prepared CF@CNS for certain time (10, 30, and 60 min) under N_2_ plasma using a 13.56 MHz radio frequency (RF) source with the power of 200 W and the pressure of 10 Pa. Note that in the following context, the CF@PN–CNS unless specifically stated represents the sample with 30 min treatment. The samples with 10 and 60 min treatment were denoted as CF@PN–CNS–10 and CF@PN–CNS–60. For investigating the effects of plasma treatment, the as‐prepared CF@CNS was calcined in the tube furnace for 30 min under N_2_ atmosphere at the temperature of 300 °C. The resultant sample was denoted as CF@N–CNS.

*Characterizations*: XRD data were collected using a Bruker D8 X‐ray diffractometer with Cu Kα radiation (λ = 0.15406 nm). X‐ray photoelectron spectroscopy (XPS) measurements were performed on a K‐Alpha1063 spectrometer with Al Kα radiation at 6 mA and 12 kV. The morphology of the as‐prepared sample was characterized by a scanning electron microscope (Nova NanoSEM). Thermogravimetric analysis was tested on an STA 449 C thermoanalyzer in air with a heating rate of 5 °C min^−1^ from 25 to 1000 °C.

*Electrochemical Measurements*: To investigate the lithium plating/stripping performance, CR2016 coin‐type cells were assembled by using CF@CNS, CF@PN–CNS, and CF@N–CNS as the working electrodes, respectively, Li foils as the counter electrode, and Celgard membranes K2045 as the separator. The electrolyte was 1 m bis‐(trifluoromethane)sulfonamide lithium salt (LiTFSI) in a mixture of 1,3‐dioxolane (DOL) and 1,2‐dimethoxyethane (DME) (v/v = 1:1) with 1 wt% LiNO_3_. A Neware CT‐3008W battery testing system was employed for the galvanostatic charge/discharge tests. The assembled cell was first cycled between 0.01 and 1 V at 0.25 mA cm^−2^ for three times to stabilize the electrode interface. For the cycling stability measurement, 1 mAh cm^−2^ of lithium was plated on the substrate at 1 mA cm^−2^ and subsequently stripped at 1 mA cm^−2^ until the cutoff potential reached 1 V (vs Li^+^/Li) in each cycle. CV measurements were performed on a CHI660D electrochemical workstation between −0.1 and 1.0 V. EIS was collected by a Princeton Parstat 2273 workstation from 10^5^ to 10^−2^ Hz. Symmetrical cells were assembled to evaluate the cycling stability and voltage hysteresis of 4 mAh cm^−2^; lithium was first plated on the substrate in half cells, and then the lithium‐plated electrodes were extracted from the half cells and the symmetrical cell was reassembled with two identical electrodes. The cells were cycled at 1 mA cm^−2^ for 1 h. For the full cell test, the as‐prepared lithium anodes (CF–Li, CF@CNS–Li, CF@N–CNS–Li, and CF@PN–CNS–Li) extracted from the half‐cell were first washed by diethyl carbonate (DEC) and then dried in the chamber of the glovebox. The LiFePO_4_ cathode was fabricated by mixing LiFePO_4_, acetylene black, and polyvinylidene difluoride (w/w = 8:1:1) with *N*‐methyl‐2‐pyrrolidone as the solvent. The cells were charged and discharged between 2.5 and 4.2 V at different current densities.

## Conflict of Interest

The authors declare no conflict of interest.

## Supporting information

Supplementary
