# Supplementary material for: Plasma‐Strengthened Lithiophilicity of Copper Oxide Nanosheet–Decorated Cu Foil for Stable Lithium Metal Anode
Source: Adv Sci (Weinh). 2019 Aug 15;6(20):1901433. doi: 10.1002/advs.201901433 (PMC6794617; doi:10.1002/advs.201901433)
Supplement: Supplementary file 1 — Supplementary [file ADVS-6-1901433-s001.pdf]

Copyright WILEY-VCH Verlag GmbH & Co. KGaA, 69469 Weinheim, Germany, 2019.

ADVANCED SCIENCE
Open Access

Supporting Information

for *Adv. Sci.,* DOI: 10.1002/advs.201901433

Plasma-Strengthened Lithiophilicity of Copper Oxide Nanosheet–Decorated Cu Foil for Stable Lithium Metal Anode

Jingyi Luan, Qi Zhang, Hongyan Yuan, Dan Sun, Zhiguang Peng, Yougen Tang, Xiaobo Ji, and Haiyan Wang**

Copyright WILEY-VCH Verlag GmbH & Co. KGaA, 69469 Weinheim, Germany, 2019.

Supporting Information

Plasma strengthened lithiophilicity of copper oxide nanosheets decorated Cu foil for stable lithium metal anode

Jingyi Luan, Qi Zhang, Hongyan Yuan, Dan Sun, Zhiguang Peng*, Yougen Tang, Xiaobo Ji, Haiyan Wang*

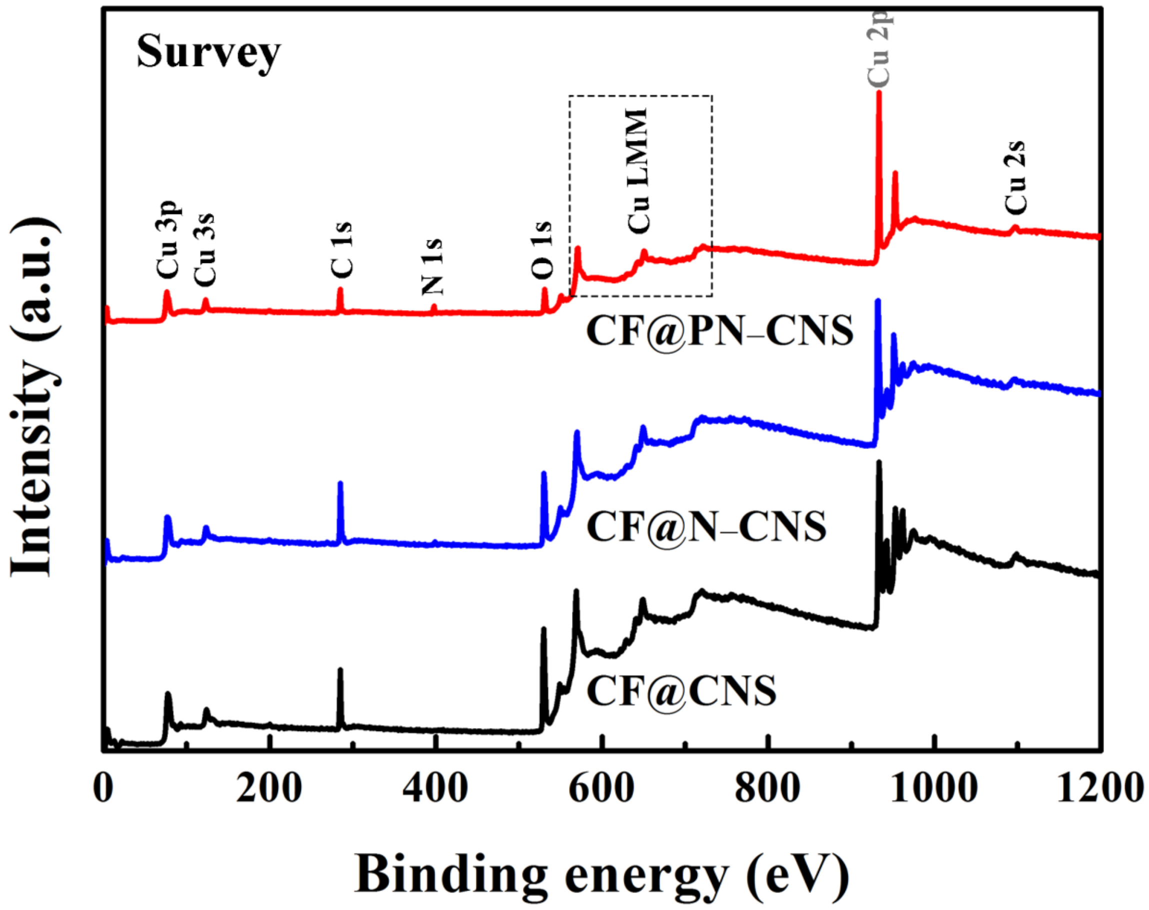

Figure S1. XPS survey spectrum of CF@CNS, CF@N−CNS and CF@PN−CNS.

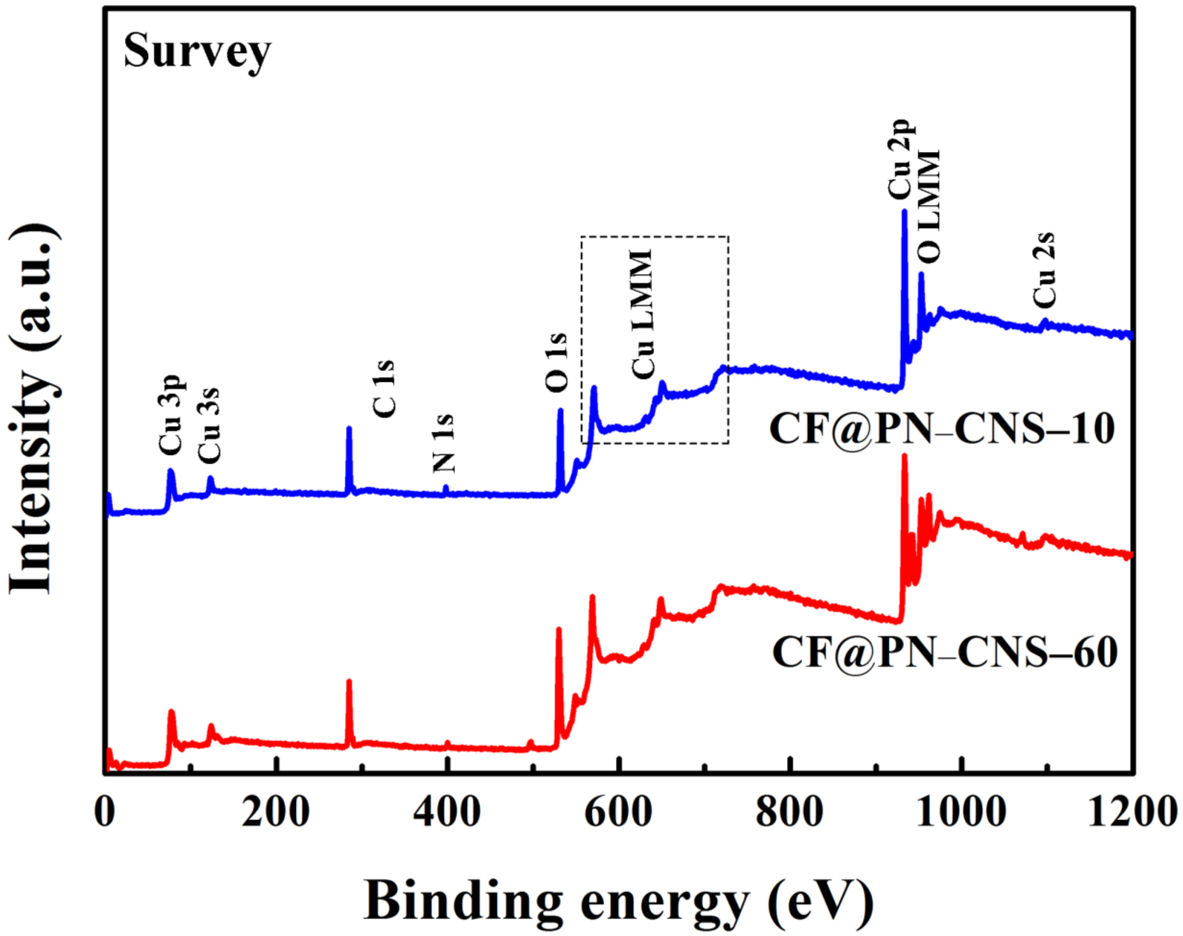

Figure S2. XPS survey spectrum of CF@PN−CNS−10 and CF@PN−CNS−60.

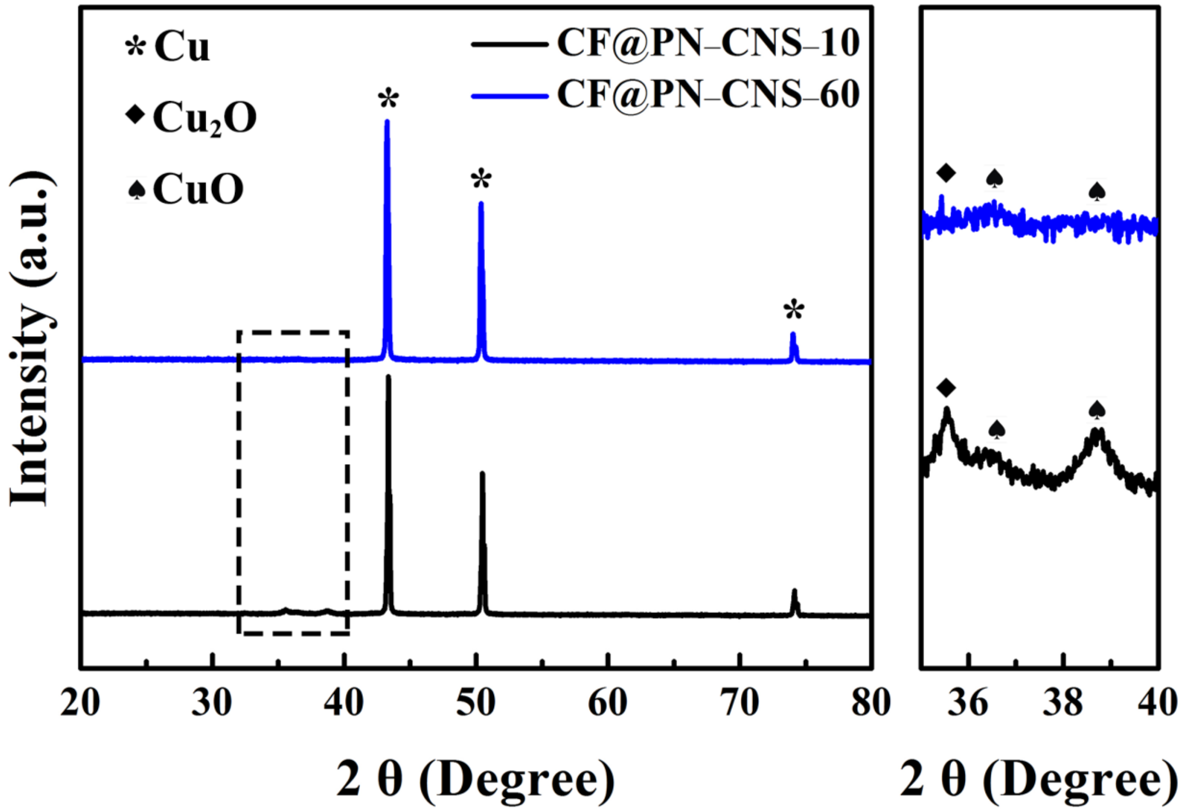

Figure S3. XRD patterns of CF@PN−CNS−10 and CF@PN−CNS−60.

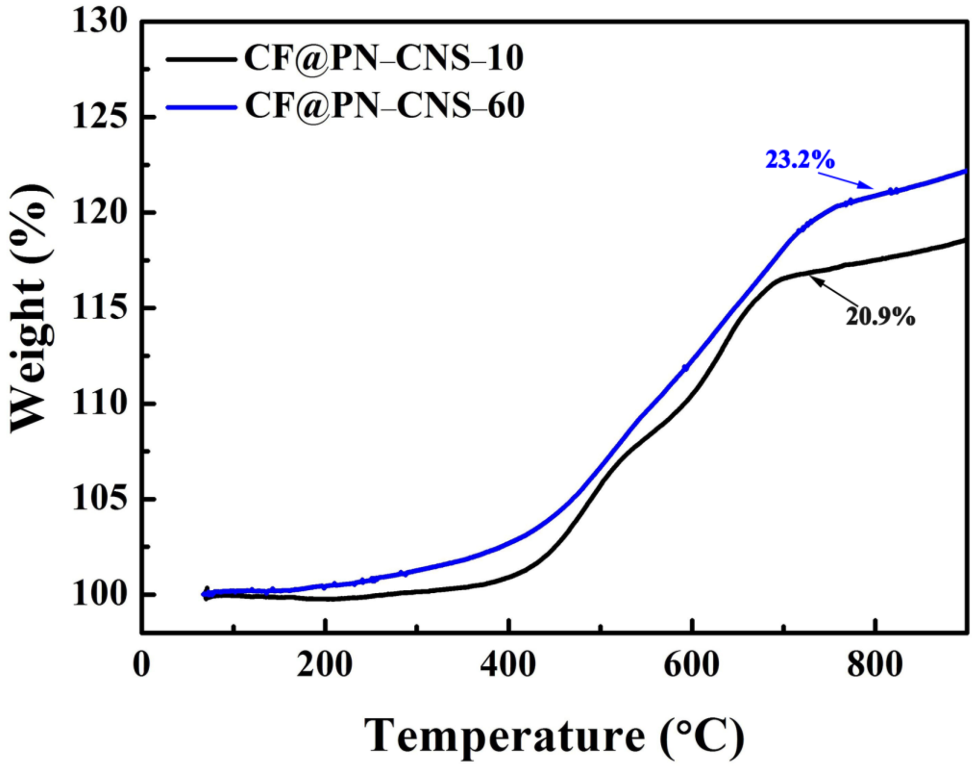

Figure S4. TG curves of CF@PN−CNS−10 and CF@PN−CNS−60.

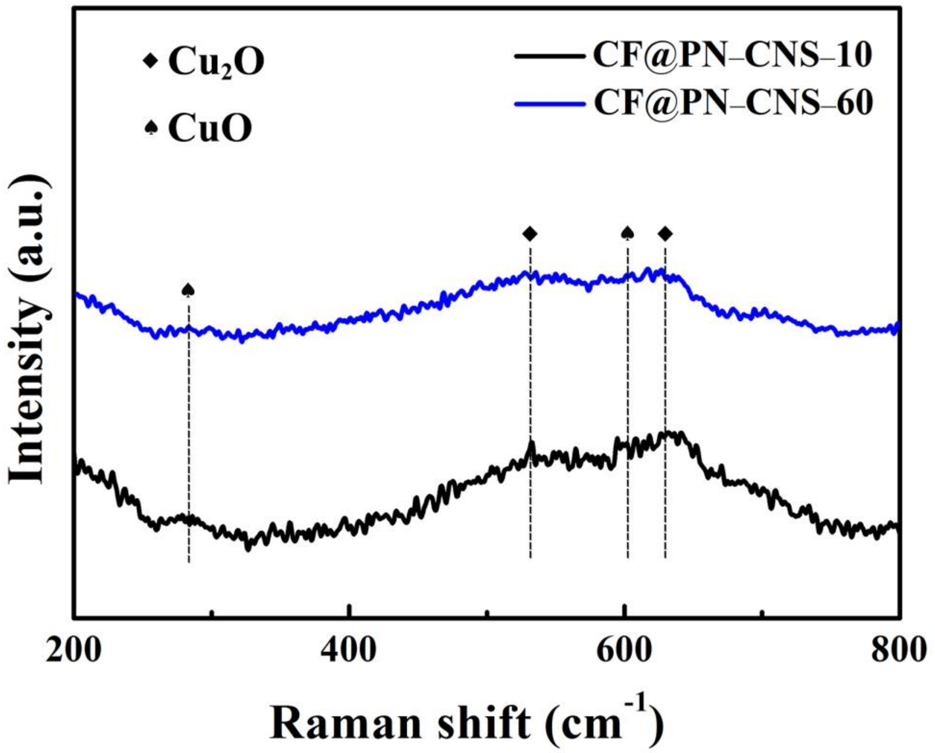

Figure S5. Raman spectra of CF@PN−CNS−10 and CF@PN−CNS−60.

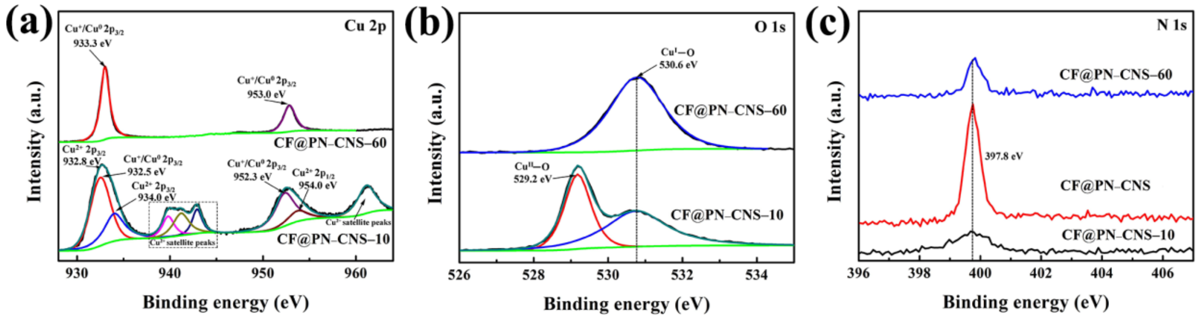

Figure S6. a) Cu 2p XPS spectrum of CF@PN−CNS−10 and CF@PN−CNS−60. b) O 1s XPS spectrum of CF@PN−CNS−10 and CF@PN−CNS−60. c) N 1s XPS spectrum of CF@PN−CNS, CF@PN−CNS−10 and CF@PN−CNS−60.

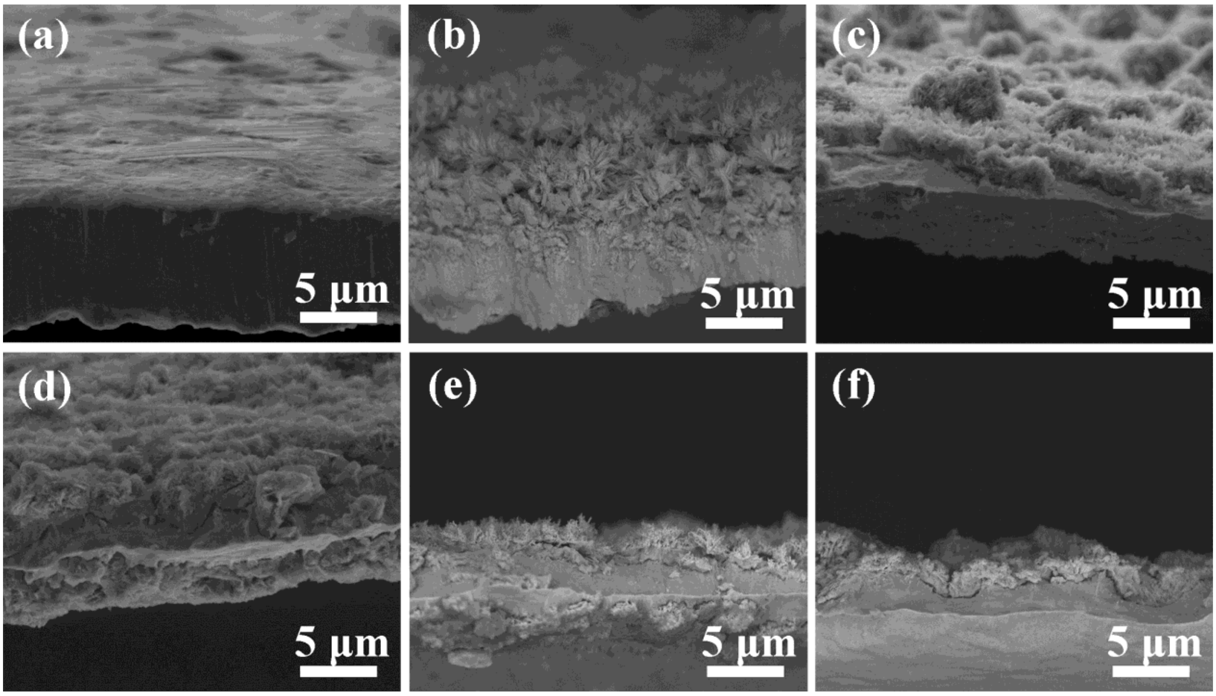

Figure S7. SEM images of a) CF, b) CF@CNS, c) CF@N−CNS, d) CF@PN−CNS−10, e) CF@PN−CNS and f) CF@PN−CNS−60.

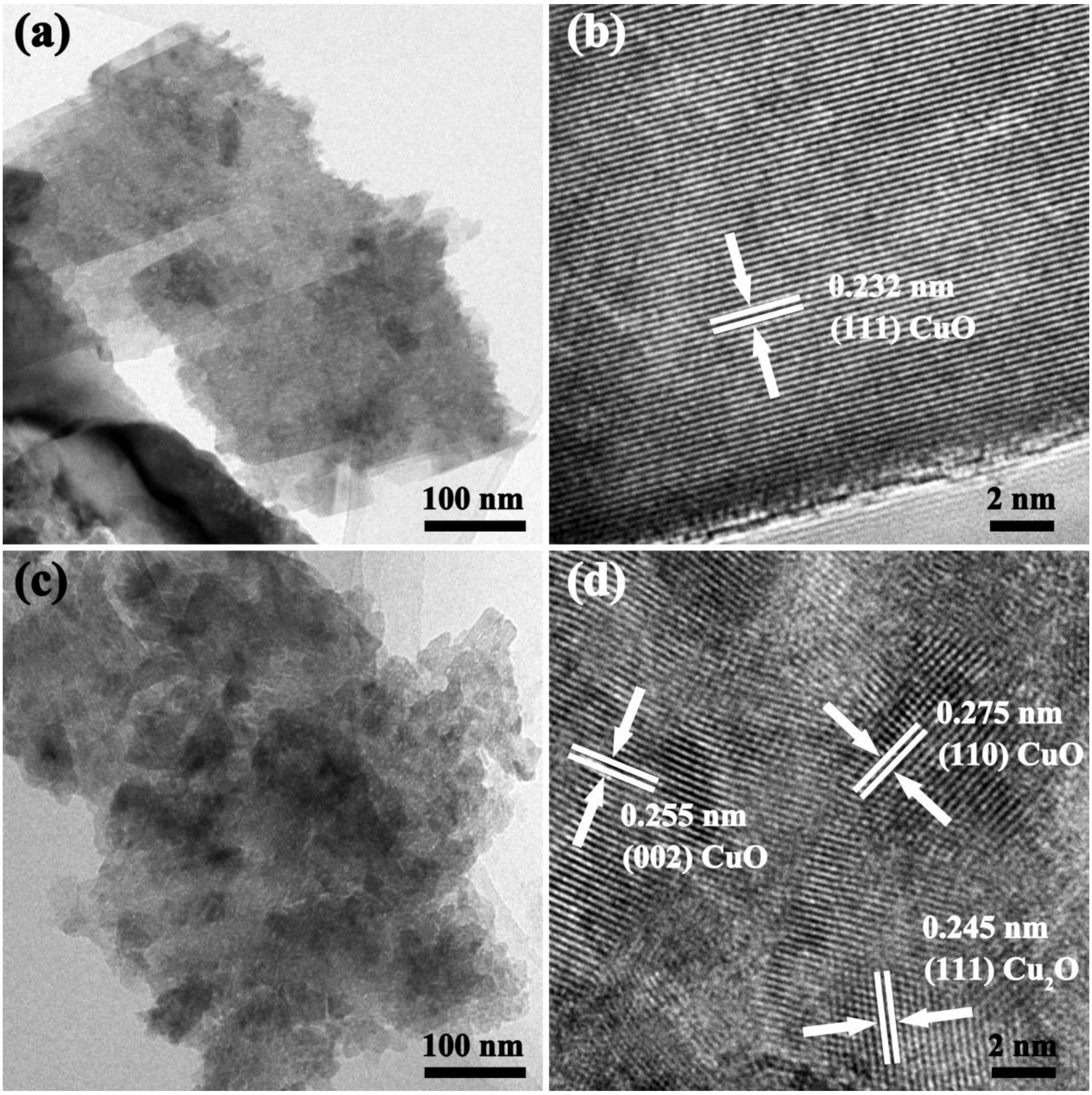

Figure S8. TEM and HRTEM images of a, b) CF@CNS and c, d) CF@PN−CNS.

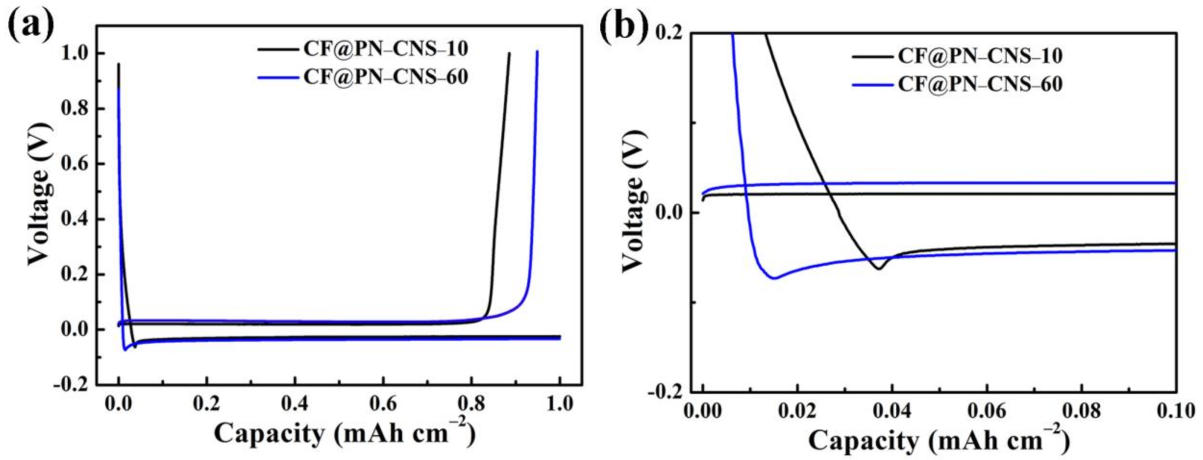

Figure S9. a) Charge−discharge curves of the plating and stripping processes at 1 mA cm^{-2} in the first cycle of CF@PN−CNS−10 and CF@PN−CNS−60. b) Magnified image of the charge−discharge curves of CF@PN−CNS−10 and CF@PN−CNS−60 during initial lithium plating.

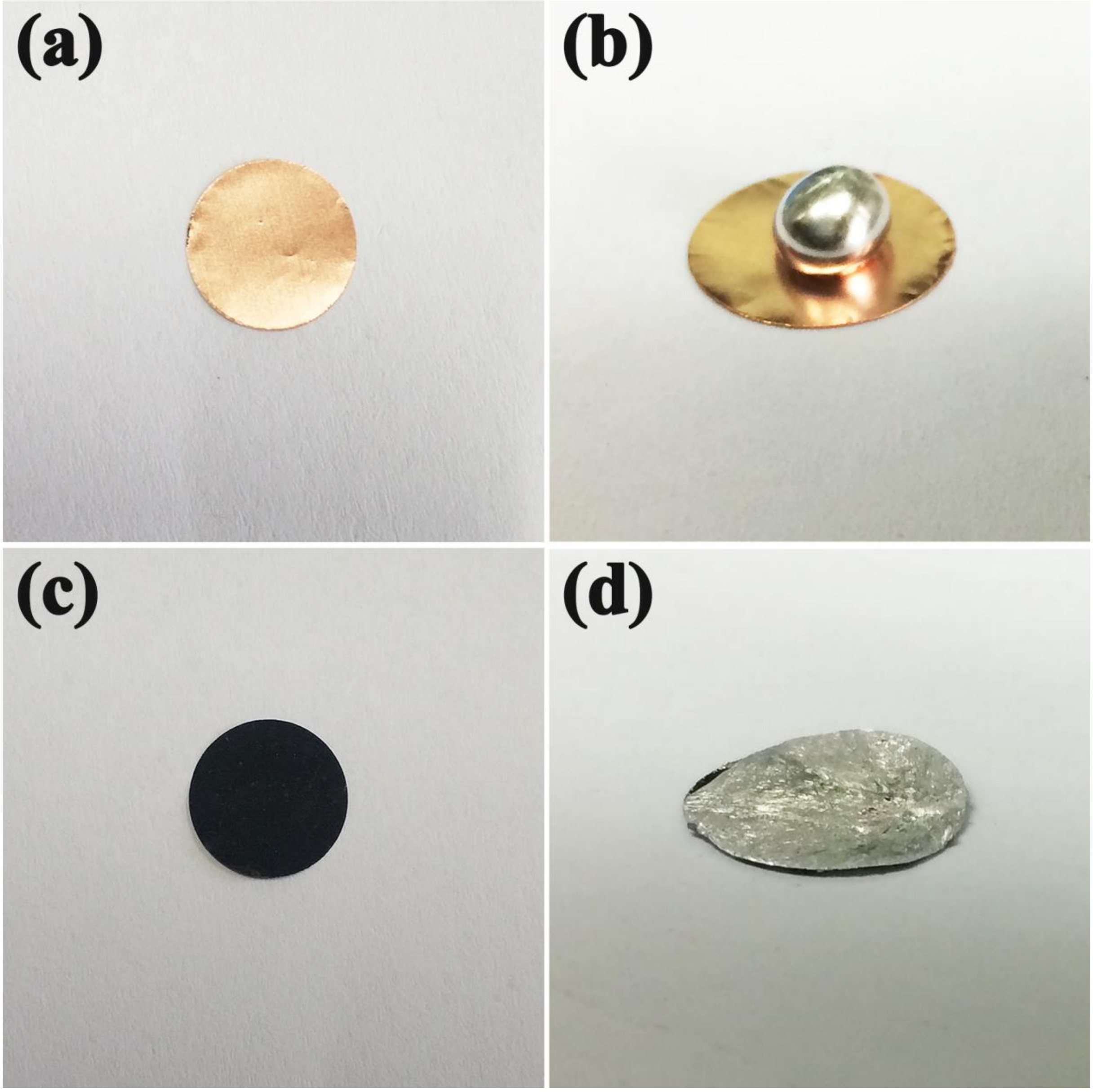

Figure S10. Photographs of a) CF and c) CF@PN−CNS. Photographs of surface wetting of molten lithium on b) CF and d) CF@PN−CNS.

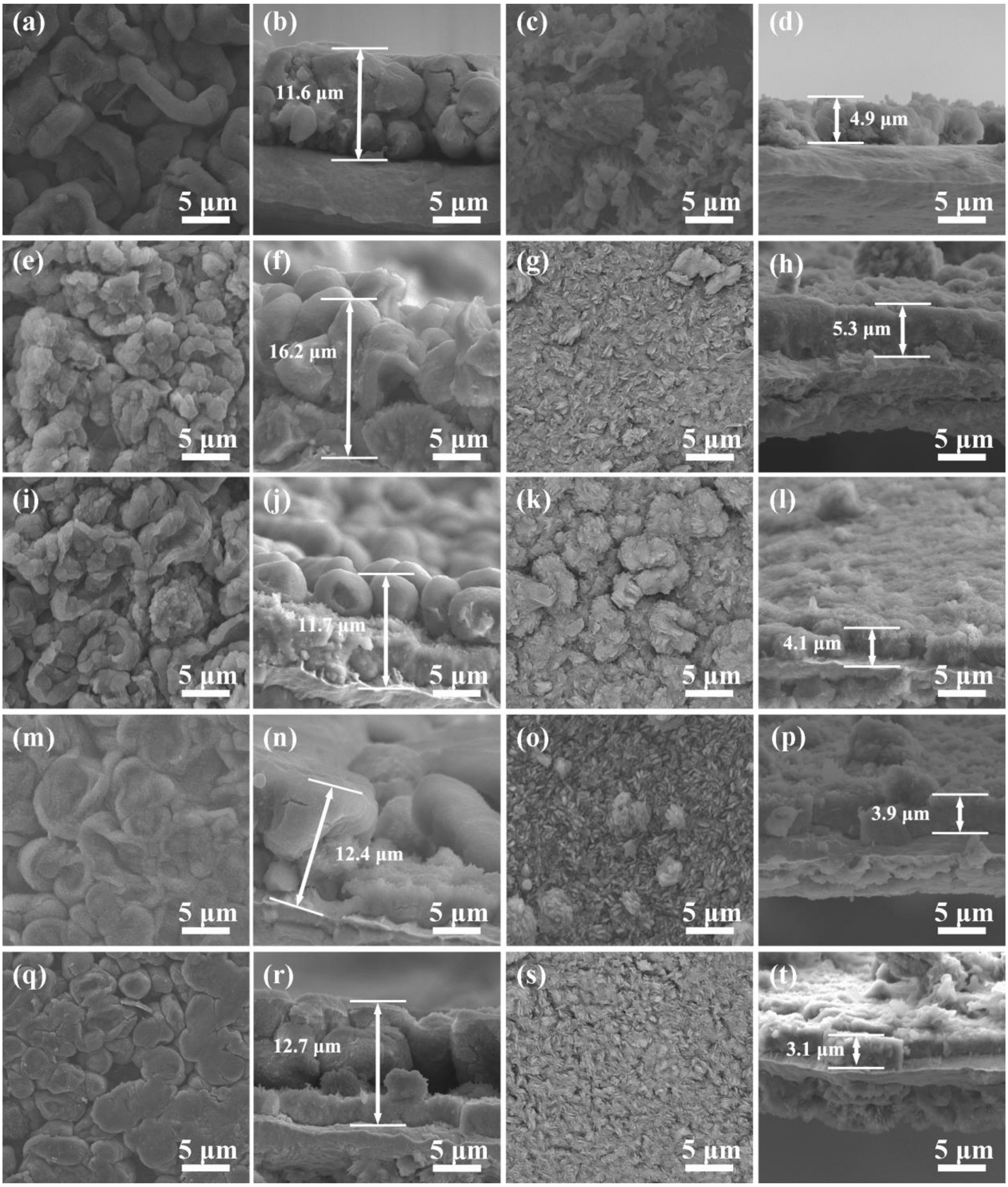

Figure S11. SEM images of a−d) CF, e−h) CF@CNS, i−l) CF@N−CNS, m−p) CF@PN−CNS−10, q−t) CF@PN−CNS−60 after the first lithium plating and the first lithium stripping.

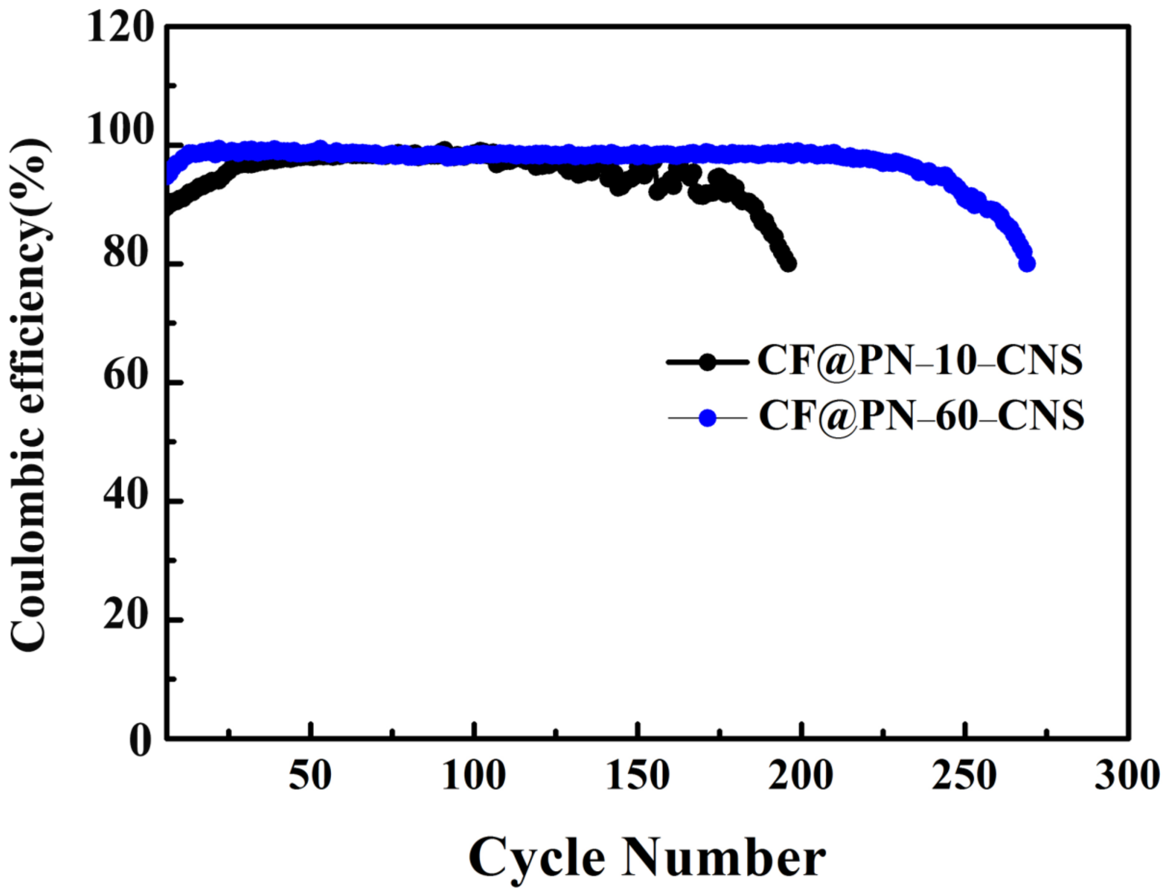

Figure S12. The comparison of Coulombic efficiency at 1 mA cm^{-2} with a specific capacity of 1 mAh cm^{-2} of CF@PN−CNS−10 and CF@PN−CNS−60.

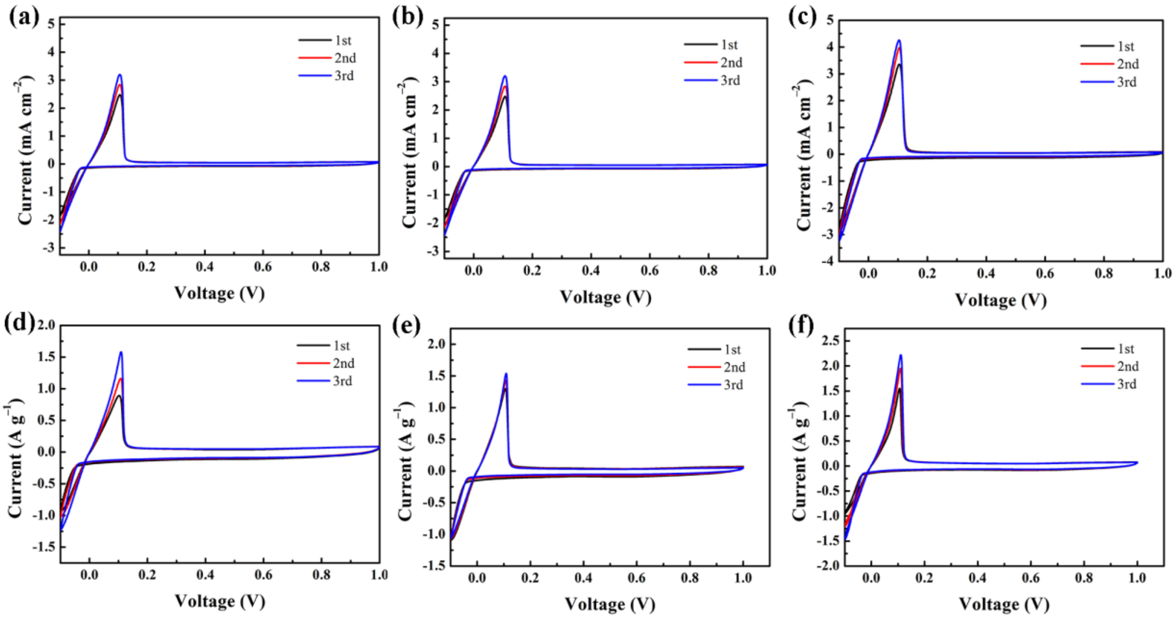

Figure S13. CV curves of a) CF, b) CF@CNS, c) CF@N−CNS, d) CF@PN−CNS−10, e) CF@PN−CNS and f) CF@PN−CNS−60 with lithium counter electrode in the initial three cycles at 0.2 mV s^{-1} between −0.1 and 1.0 V

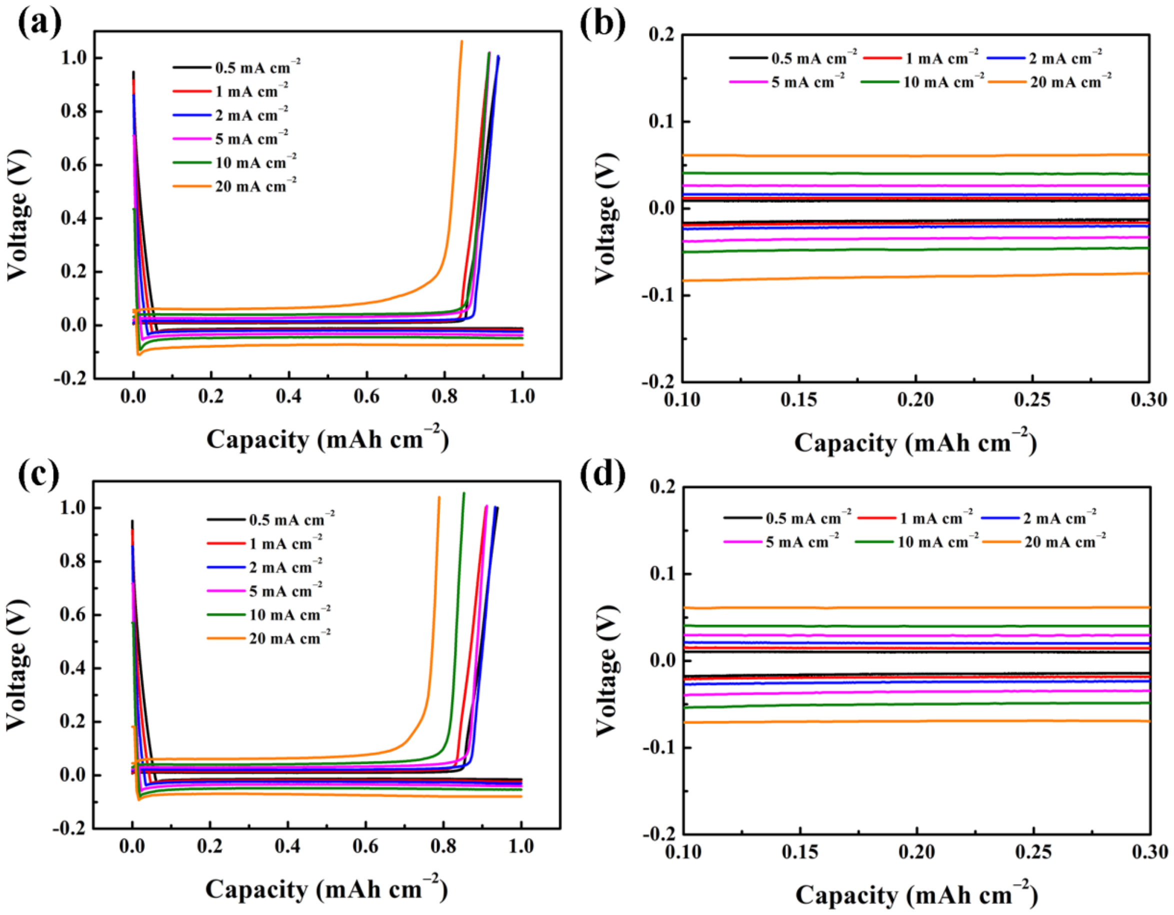

Figure S14. Plating/stripping profiles of a) CF@PN−CNS−10 and c) CF@PN−CNS−60 at various current densities. Magnified image of the plating/stripping profiles of b) CF@PN−CNS−10 and d) CF@PN−CNS−60 measured from 0.1 to 0.3 mAh cm^{-2}.

**Materials**	**Current density**	**Cycle number**	**Coulombic efficiency**	**Ref.**
MnO_2−Cu	0.5 mA cm^{-2}	150	97%	1[1]

ZnO decorated three-dimensional hierarchical porous carbon	1 mA cm^{-2}	200	97.1%	2[2]
Cu-CuO-Ni hybrid structure	1 mA cm^{-2}	250	95%	3[3]
CuO arrays on Cu collector	0.5 mA cm^{-2}	180	94%	4[4]
Three-dimensional porous Cu	0.5 mA cm^{-2}	200	98%	5[5]
Nanoporous/macroporous structure Cu current collector	1 mA cm^{-2}	200	98%	6[6]
Three-dimensional Cu skeleton	0.5 mA cm^{-2}	50	97%	7[7]
Graphene anchored Cu foam	2 mA cm^{-2}	150	97.4%	8[8]
Plasma-treated copper oxide nanosheets decorated Cu foil	1 mA cm^{-2}	500	99.6%	This work

Table S1. Comparison of the electrochemical performance of several materials.

[1] M. Hu, Y. Yuan, M. Guo, Y. Pan, D. Long, *Journal of Materials Chemistry A* **2018**, *6*, 14910.

[2] C. Jin, O. Sheng, J. Luo, H. Yuan, F. Cong, W. Zhang, H. Hui, Y. Gan, X. Yang, L. Chu, *Nano Energy* **2017**, *37*, 177.

[3] S. Wu, Z. Zhang, M. Lan, S. Yang, J. Cheng, J. Cai, J. Shen, Y. Zhu, K. Zhang, W. Zhang, *Advanced Materials* **2018**, *30*, 1705830.

[4] C. Zhang, W. Lv, G. Zhou, Z. Huang, Y. Zhang, R. Lyu, H. Wu, Q. Yun, F. Kang, Q.-H. Yang, *Advanced Energy Materials* **2018**, *8*, 1703404.

[5] Y. Shi, Z. Wang, H. Gao, J. Niu, W. Ma, J. Qin, Z. Peng, Z. Zhang, *Journal of Materials Chemistry A* **2019**, *7*, 1092.

[6] H. Liu, E. Wang, Q. Zhang, Y. Ren, X. Guo, L. Wang, G. Li, H. Yu, *Energy Storage Materials* **2019**, *17*, 253.

[7] C. P. Yang, Y. X. Yin, S. F. Zhang, N. W. Li, Y. G. Guo, *Nature Communications* **2015**, *6*, 8058.

[8] G. Yang, J. Chen, P. Xiao, P. O. Agboola, I. Shakir, Y. Xu, *Journal of Materials Chemistry A* **2018**, *6*, 9899.
